# Dynamic inking of large-scale stamps for multiplexed microcontact printing and fabrication of cell microarrays

**DOI:** 10.1371/journal.pone.0202531

**Published:** 2018-08-23

**Authors:** Julie Foncy, Aurore Estève, Amélie Degache, Camille Colin, Xavier Dollat, Jean-Christophe Cau, Christophe Vieu, Emmanuelle Trévisiol, Laurent Malaquin

**Affiliations:** 1 LAAS-CNRS, Université de Toulouse, CNRS, INSA, Toulouse, France; 2 Innopsys, Parc d’activités Activestre, Carbonne, France; Basque Center for Materials, Applications and Nanostructures, PORTUGAL

## Abstract

Microcontact printing has become a versatile soft lithography technique used to produce molecular micro- and nano-patterns consisting of a large range of different biomolecules. Despite intensive research over the last decade and numerous applications in the fields of biosensors, microarrays and biomedical applications, the large-scale implementation of microcontact printing is still an issue. It is hindered by the stamp-inking step that is critical to ensure a reproducible and uniform transfer of inked molecules over large areas. This is particularly important when addressing application such as cell microarray manufacturing, which are currently used for a wide range of analytical and pharmaceutical applications. In this paper, we present a large-scale and multiplexed microcontact printing process of extracellular matrix proteins for the fabrication of cell microarrays. We have developed a microfluidic inking approach combined with a magnetic clamping technology that can be adapted to most standard substrates used in biology. We have demonstrated a significant improvement of homogeneity of printed protein patterns on surfaces larger than 1 cm^2^ through the control of both the flow rate and the wetting mechanism of the stamp surface during microfluidic inking. Thanks to the reproducibility and integration capabilities provided by microfluidics, we have achieved the printing of three different adhesion proteins in one-step transfer. Selective cell adhesion and cell shape adaptation on the produced patterns were observed, showing the suitability of this approach for producing on-demand large-scale cell microarrays.

## Introduction

Cell-based assays are widely used for drug screening, profiling applications [1,2] tissue engineering and fundamental biological studies [3,4]. They can allow rapid identification of genetic determinants of disease, discovery of cellular function modulators and probing of complex and dynamic relationships between cells and their environment [5]. Miniaturization and parallelization of such assays, known as cell microarrays, provide critical advantages in comparison to microtiter plates such as increased throughput for high content screening purposes, small reagent volumes and larger range of detection methods [2].

Micro-patterns of extra cellular matrix (ECM) proteins can be used to control cell adhesion or to study cell differentiation or motility on a substrate [6,7]. This approach is relatively straightforward but requires the development of patterning techniques offering a resolution at the cell level together with the capacity to process large surfaces with high reproducibility and homogeneity. In this paper, our aim is to address this capability by exploiting the capacity of microcontact printing technique to produce high resolution patterns while extending its reproducibility and capacity in the processing of large areas typically above 10 cm^2^.

Microcontact printing (μCP) was first introduced by Xia *et al*. [8] in 1996. It is based on a printing principle involving an elastomeric stamp, an ink and a receiving solid substrate. This rather simple and cost-effective technology can produce micro- and nano-patterns made of a large range of biomolecules [9] and nanoparticles [10]. The efficiency of μCP and its compatibility with a large range of inks has been demonstrated but so far, most validations with homogeneous deposition were obtained on relatively small surfaces. In the literature, typical homogeneous printed areas are ranging up to 300 μm × 300 μm [9,11–13]. Behind the objective of using microcontact printing process as a patterning technique for cell microarray fabrication, thus lies the processing of significantly larger surfaces together with the improvement of the deposition homogeneity required for large-scale manufacturing. Beyond the uniformity of the stamp-surface contact during the printing step, one of the major source of process variability is related to the inking step and to the uneven distribution of the adsorbed ink molecules at the stamp surface. Conventional approaches such as droplet-based inking, if suitable for small-area functionalization appears to be inappropriate for large surface processing [14]. The purpose of this work focuses on an alternative method for stamp inking to improve the quality of the printed patterns over large areas. This concept combines the reproducibility of microfluidics for the control of the inking process with the use of magnetic forces to first, reversibly clamp the microfluidic device to the topographically structured stamp during inking and then, to ensure a uniform contact between the inked stamp and the substrate during printing. This approach provides an accurate control of the incubation parameters, wetting and dewetting mechanisms, which turned out to be essential in the inking step where the control of the liquid meniscus velocity over the micro-structured PDMS stamp is critical. Based on this concept, we developed several microfluidic designs with independent channels to multiplex the inking step with different biomolecules and to create patterns of different compositions in one single print. This approach benefits from the multiplexing and automation capabilities offered by microfluidics and uses a simple and reliable clamping technique which is compatible with almost all type of stamp designs. It differs from previously reported multiplexing methods that require sequential printing steps [9], that involves complex fabrication processes [15] or require expensive ink deposition systems [16]. More simple approaches based on multilevel stamps were also investigated [17] but limitations in the printed areas and pattern shapes imposed by the inking step impedes patterning over large areas. Methods based on microfluidic inking were also proposed in literature but so far, these methods were limited to gradient patterns [18].

To validate the potentiality of this new optimized process, we addressed the fabrication of cell microarrays by printing patterns of extra cellular matrix (ECM) proteins generated to control the localization of adherent eukaryotic living cells in well-registered positions over typical 2D cell culture supports.

## Material and methods

**Fig 1:**
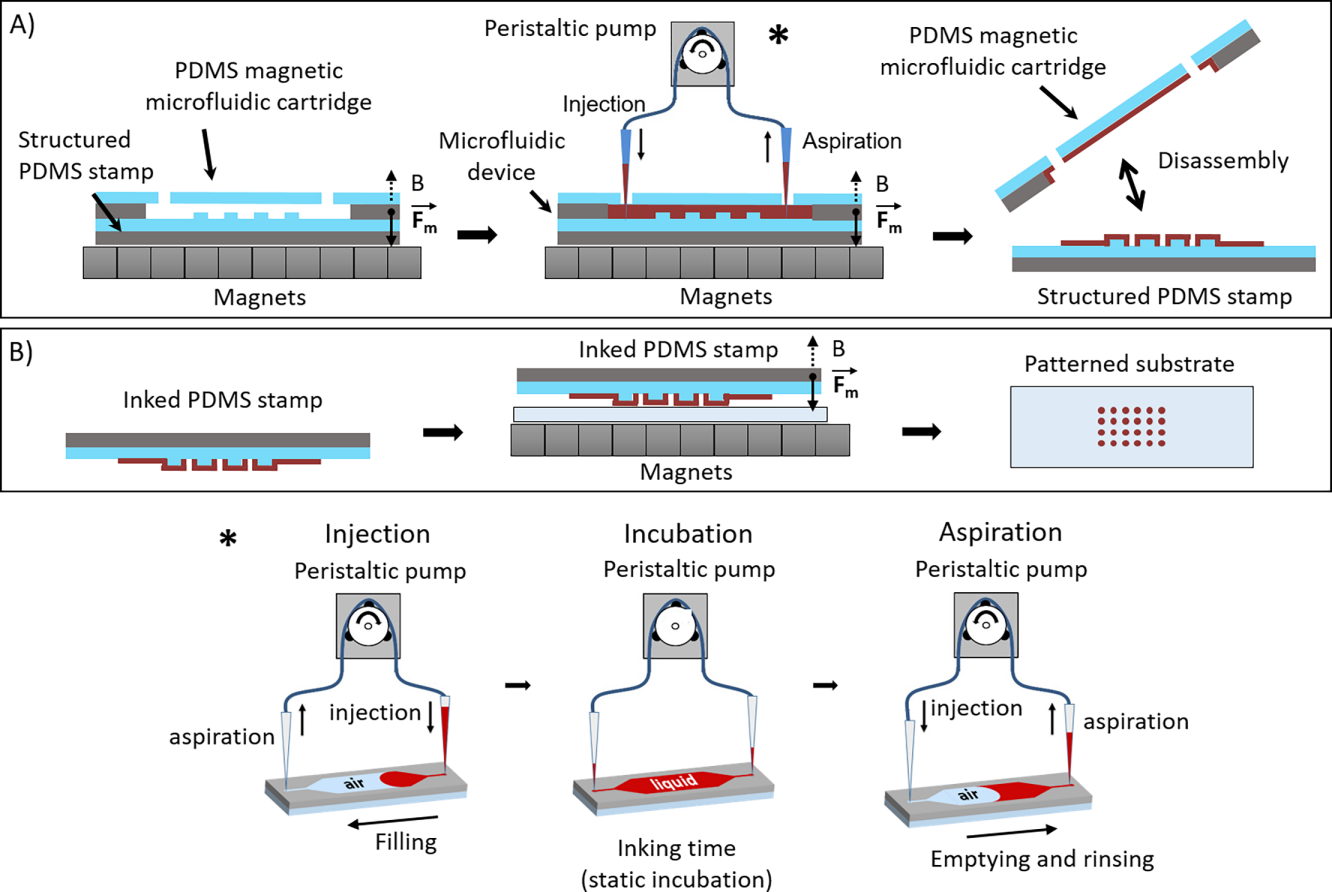
**Schematic description of the magnetic microcontact printing workflow**. (A) Microfluidic inking of a structured elastomeric magnetic stamp. The star symbol redirects to an illustration of the three steps involved in microfluidic inking. (B) Magnetic driven uniform contact between the inked magnetic stamp and the substrate.

Fig 1 illustrates the general procedure that we implemented for the inking of a PDMS stamp using a reversible microfluidic device. The different parts of the process are detailed below and refer to this figure.

### Magnetic fluidic device design and fabrication

#### Device design

Three different microfluidic device designs (S1 Appendix) adapted to three different chamber areas or channel(s) were investigated. The chamber dimensions of design 1 were 10 mm long x 10 mm wide x 0.3 mm high, thus providing an inking area of 1 cm^2^. Access to the chamber was provided by two channels (10 mm long x 0.5 mm wide). Second design was designed for the inking of larger surfaces typically corresponding to conventional glass slides. Dimensions of the chamber were set to 60 mm long x 20 mm wide x 0.3 mm high. Similar to first design, two channels (12 mm long x 0.5 mm wide) joining inlet and outlet were integrated to provide access to the fluidic chamber. The third design was dedicated to multiplexed inking. It was designed with six independent channels with a length of 50 mm, a width of 2 mm and a height of 0.4 mm.

#### Fabrication

Aluminium master molds (aluminium 2017A) were manufactured by micro-milling using a 500-μm-diameter mechanical flat-end mill. Microfluidic devices were fabricated in two steps (see S2 Appendix) by sequentially pouring two layers of PDMS mixture on the aluminium mold. PDMS prepolymer and curing agent (Sylgard 184, Dow Corning) were first mixed in a 10:1 weight ratio. Iron powder (Sigma Aldrich, hydrogen reduced, 50 μm diameter) was further added to the PDMS prepolymer mixture with a loading of 1:1 (w:w). The magnetic PDMS prepolymer solution was degassed and carefully poured onto the master. An elastomeric blade was used to remove excess of magnetic PDMS on top of the protruding structures of the master mold. The layer was further cured for 1 hour at 65°C leading to a 300 μm thick magnetic PDMS layer (or 400 μm thick magnetic PDMS layer for the multiplexed design). Then, a second layer of native PDMS was casted on top of the magnetic layer and cured (12 h, 65°C). Finally, PDMS microfluidic devices were removed from the mold and inlets and outlets were punched. This two-step approach is highly advantageous as it combines the magnetic properties of the devices for reversible clamping with the integration of fully transparent microfluidic channels.

### Magnetic stamp design and fabrication

Magnetic PDMS stamps were structured with a variety of feature sizes (from 10 to 150 μm) and forms (squares, lines, spots and triangles). SU-8 resist master molds required for the fabrication of micro-structured PDMS stamps were fabricated by photolithography. After UV exposure of the resist layer (SU-8 3050, thickness of 25 μm), the master mold was developed in SU-8 developer for 9 minutes and rinsed using isopropanol. Then a PerFluorodecylTrichloroSilane (PFTS) coating was performed by spray pyrolysis deposition (SPD) to confer anti-adhesive properties to the mold surface [19]. Similar to the fabrication of the microfluidic devices, structured magnetic PDMS stamps were obtained in two steps. A thin layer of degassed PDMS solution (mixture of PDMS prepolymer and curing agent in 10:1 weight ratio) was poured onto the master mold and cured for 1 hour at 65°C. Magnetic PDMS solution (50/50, w/w) of iron powder (Sigma Aldrich, hydrogen reduced, 50 μm diameter) mixed with PDMS solution, was degassed and casted on top of the first layer and cured for 12 hours at 65°C. Finally, the structured PDMS stamps were removed from the mold.

### Microcontact printing (μCP) process

#### Droplet-based inking

As a reference, a manual inking step of the structured stamps was performed by deposition of a drop of ink on the structured surface of a magnetic PDMS stamp. The process was performed according to our previously published work [20]. A volume of 1 mL of the inking solution (Cy3- and Cy5- labelled streptavidin (Thermo Fisher) at 1μg.ml^-1^ in PBS 1x or fibronectin from bovine plasma (Sigma Aldrich) at 20μg.ml^-1^ in PBS 1x) was used. After an incubation step of 1 minute, the drop was removed by aspirating with a pipette and the stamp surface was washed with phosphate-buffered saline (PBS 1x, Gibco) and blown dried with nitrogen.

#### Microfluidic inking

Magnetic microfluidic devices were manually aligned and brought into contact with the magnetic PDMS stamps. Reversible clamp between the microfluidic device and the stamp was ensured by positioning the whole device on an array of 8 x 8 magnets (15 mm x 15mm x 15 mm, Neodyme Ferbore). The magnetic field generated by the permanent magnet array provides enough strength to hold the stamp and the microfluidic device together, and provides an efficient sealing all along the experiments (no leakage was observed for input pressures up to 150 mbar). The microchannel aspect ratio was kept above 1:50 (height: width) to prevent potential collapse of the structures. Disassembly of the two parts was easily done by moving magnets away. The inking step was performed using a system integrating a peristaltic pump connected to both inlet and outlet of the microfluidic device. This allows simultaneous injection in one side and aspiration in the other side with a direct control of the flow rate in the range of 5 μL.s^-1^ to 100 μL.s^-1^. The ink (Cy3- and Cy5- labelled streptavidin (Thermo Fisher) at 1μg.ml^-1^ in PBS 1x or fibronectin from bovine plasma (Sigma Aldrich) at 20μg.ml^-1^ in PBS 1x) was loaded from a titration plate and injected into the microfluidic device. Once the chamber and channels were filled with ink, the flow was stopped for an incubation time of one minute. The ink was then removed from the fluidic channel, using the same liquid handling system. A rinsing step using PBS 1x (Gibco) was performed in the same way. For multiplexed microfluidic devices, the same procedure was applied sequentially to each independent channel. Before separation, channels and chambers were emptied by simultaneous air injection and liquid aspiration using the same peristaltic pump system. Finally, magnets were removed to separate the stamp from the microfluidic device and the inked stamp was finally dried under a stream of nitrogen.

#### Printing on glass substrates

The printing process was performed according to previously published methods [19]. Briefly the inked stamps were brought into contact with a glass slide that was previously activated through air plasma treatment (Diener Pico, 50 W, 0.5 mBar, 1 min 30 sec). Contact time was set to 1 minute. Magnets were placed beneath the glass slide in order to ensure a uniform contact between the magnetic stamp and the receiving glass substrate. The adjustment of the distance between the magnet and the magnetic stamp was set for providing a homogeneous magnetic pressure of 0.2 kPa, which was identified as the optimal one for uniform contact without structure collapse. Finally, the stamp was removed and the glass slide was ready for characterization and cell culture.

### Image acquisition

Images of patterns were acquired using InnoScan 1100 AL fluorescence scanner (Innopsys) through simultaneous acquisition at 3 different wavelengths (488 nm, 532 nm and 635 nm). The fluorescence images were analysed for quantification of fluorescence intensity using Mapix software (Innopsys) or ImageJ software. Coefficients of variation (CV) were calculated as the quotient of standard deviation over the mean of fluorescence intensities. Images of cells were acquired by confocal microscopy (Zeiss, LSM 710, 63 x objective).

### Cell microarray conception

Plasma cleaned (Diener Pico, 50 W, 0.5 mBar, 1.5 min) glass slides were patterned with a protein promoting cell adhesion (fibronectin, 100 μg/mL, Sigma Aldrich, in PBS 1x, pH 7.4), using the microcontact printing procedures described above. PDMS stamps were structured with various features (squares, lines, spots and triangles) with sizes and spacing ranging from 10 μm to 50 μm. A solution of PLL-g-PEG (1 mg/mL, SuSoS, PLL(20)-g[3.5]-PEG(2) in PBS 1x, pH 7.4) was then incubated for 30 minutes to prevent cell adhesion between the patterns. After rinsing (PBS 1x, three times), PC3-GFP cells (prostate cancer cell line modified to produce Green Fluorescence Protein) were seeded (800,000 cells per slide). Cells were cultivated in RPMI culture medium (Gibco, with 10% Bovin Foetal Serum, 1% penicillin/streptomycin and 1% geneticin) at 37°C for 3 hours. Cell nuclei were labelled with Draq5™ fluorescent DNA dye at 5 μM (Biostatus limited, 3 min incubation at 37°C, PBS 1x rinsing three times). Finally, cells were fixed using a solution of formalin (Sigma Aldrich, 20 min incubation, PBS 1x rinsing three times) followed by dehydration with successive baths of ethanol diluted in DI water (50% ethanol, 75% ethanol and 100% ethanol, 3 minutes each).

## Results and discussion

### Protein deposition homogeneity using microfluidic inking

The impact of the microfluidic inking step was investigated by comparison to a manual droplet-based inking technique. The main evaluation criterion was the uniformity of the fluorescence intensity emitted by the patterns composed of fluorescently labelled protein. Structured stamps protruding the same features were inked using either droplet-based deposition or microfluidic inking approach using the parameters previously described in material and methods section. Protein transfer on glass slides was analyzed through fluorescence scanner (InnoScan 1100 AL, Innopsys).

**Fig 2:**
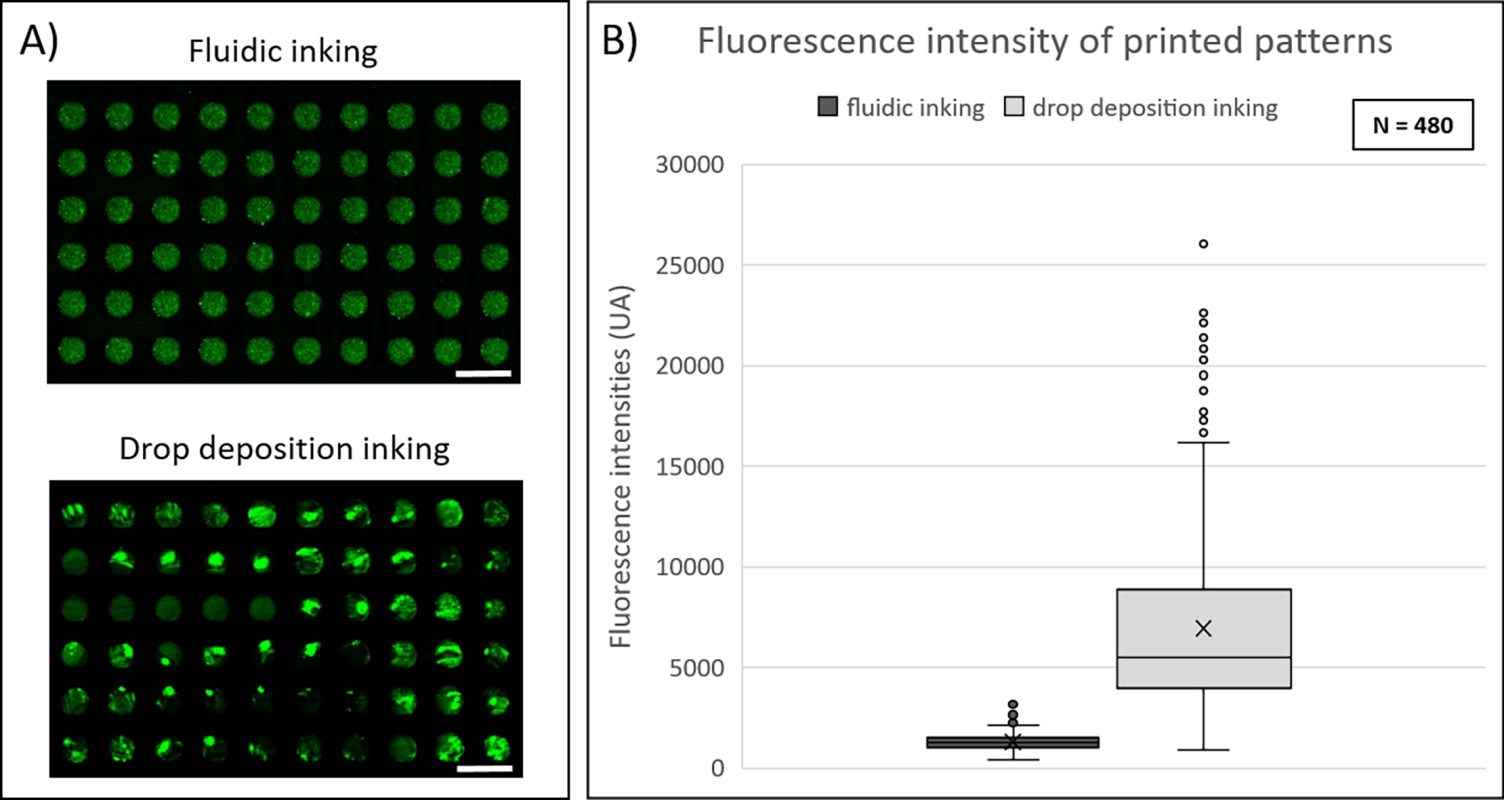
**Comparison of protein deposition homogeneity using fluidic or conventional droplet-based inking approaches**. (A) Fluorescence images of C3-labeled streptavidin patterned depositions obtained by μCP with fluidic or droplet-based inking approach. Images were obtained by fluorescence scanner InnoScan1100AL (PMT 532nm: 30%, Brightness: 40%, Contrast: 20%, resolution: 3μm/pixel). Scale bar: 200μm. (B) Fluorescence intensity values of protein depositions for both inking approaches.

In the case of a manual droplet-inking process, images showed a large dispersion in the fluorescence signal. Bright green areas corresponding to accumulation of labelled protein leading to increase of the fluorescent signal were frequently observed on the printed sample (Fig 2A). This phenomenon was not observed when microfluidic inking was used. To compare the fluorescence intensity variation from feature to feature, the mean intensity values of 480 individual features (100 μm-diameter circular spots) were measured (Fig 2B). We observed that the distribution of fluorescence intensities was more uniform when inking was performed by microfluidic approach. Indeed, a coefficient of variation of 0.3 was observed for microfluidic inking process against 0.64 for droplet-based inking process.

It is worth noticing that in our work for both conditions, patterns were printed with a magnetic assisted contact between the inked stamp and the substrate that is known to reduce significantly sources of variability induced by the printing process itself. The improvement of uniformity achieved with the microfluidic inking can thus be unambiguously attributed to the optimization of the inking step. Moreover, no significant difference in the fluorescence intensity was observed between features for any location in the fluidic chamber (S3 Appendix).

Moreover, lower volumes of inking solutions were needed in case of the microfluidic inking compared to the droplet-based inking technique. The working volumes used to fill the microfluidic chambers and tubing were 120 μl for the 1 cm^2^ microfluidic chamber and 540 μl for the chamber providing a printed area of 2 cm x 6 cm. In the case of droplet-based inking technique, the volumes of the drops necessary to ink surfaces of 1 cm^2^ and 12 cm^2^ were 500 μl and 6 ml respectively.

All together, these results suggest that the combination of microfluidic inking and magnetic-assisted printing allows to produce micro-patterns of proteins over large areas with unprecedented homogeneity.

### Optimization of the microfluidic inking parameters

First investigations were devoted to the optimization of the liquid handling in the microfluidic devices. The inking workflow relies in three subsequent steps dedicated respectively to the injection of the ink, the incubation at zero flow rate and finally the aspiration of the injected liquid at a defined flow rate. Both injection and aspiration steps involve the displacement of a three-phase contact line (air, liquid, solid) in partially filled devices. In this respect, the use of a peristaltic pump connected to both inlet and outlet channels provides unique advantages to control the meniscus velocity of the liquid phase independently of the compressibility of the air phase that may cause fluctuations of the flow rate. This approach was found to be relevant to maintain the meniscus velocity in a controlled operating range and to lower the risk of meniscus breakup caused by pinning effects on the stamp patterns. More importantly, it has been reported that the velocity of the liquid meniscus is a key parameter in the deposition of molecules or colloids in capillary assembly processes [21–23]. The movement of the contact line acts both on the contact angle value and on the mass transfer mechanisms taking place at the contact line that in return, control the deposition rate on the stamp surface. Previous work published by Fredonnet *et al*. [14] already described a dynamic inking approach with an adequate control of the meniscus displacement during the inking step, which also turned out to improve significantly the homogeneity of microcontact printing process.

**Fig 3:**
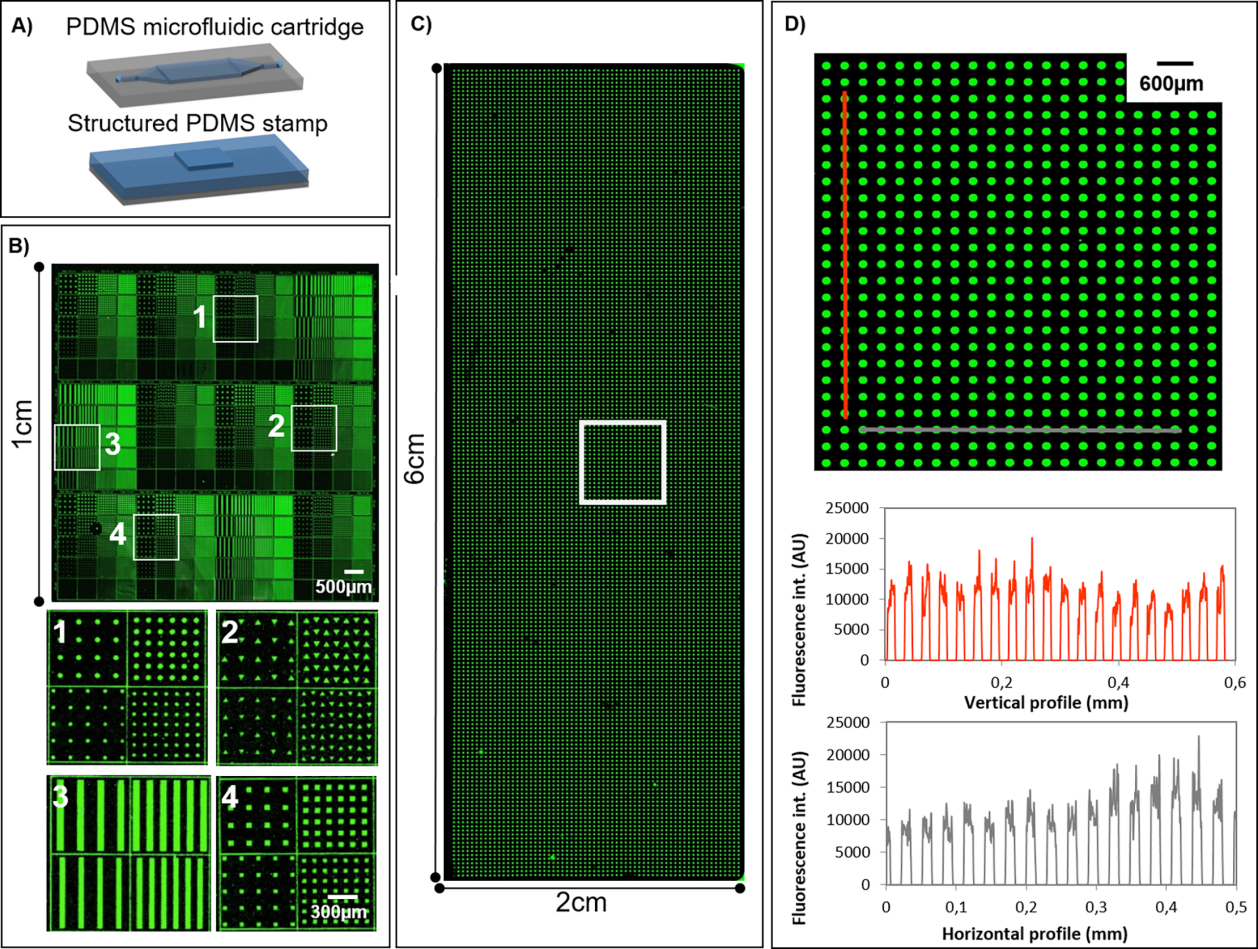
**Large-scale protein patterning**. (A) Representative scheme of microfluidic cartridge and structured stamp for fluidic inking of μCP. (B) Fluorescence image of Cy3-labeled streptavidin deposition following patterns of different sizes and shapes. Zoomed images of different areas of the deposition. (C) Fluorescence image of Cy3-labeled streptavidin patterned deposition covering a whole microscope glass slide. (D) Zoomed image and charts of vertical and horizontal fluorescence intensity profiles. Images were obtained by fluorescence scanner InnoScan1100AL (PMT 532 nm: 40%, Brightness: 50%, Contrast: 50%, resolution: 5μm/pixel).

We first observed that maintaining a constant meniscus velocity in particular during the ink withdrawal was essential to enhance the homogeneity of the molecular deposition. Fig 3A and 3B show the patterning of Cy3-labelled streptavidin in an area of 1 cm^2^ under optimized conditions. Patterns of different sizes and spacing (from 10 μm up to 50 μm) dimensions and shapes (squares, lines, circles and triangles) were used, leading to a deposition with a coefficient of variation of the fluorescence intensities around 0.3 for the whole printed area. This value is comparable to the standard values obtained with spotting techniques for the fabrication of bioassays [24]. An even larger surface of 2 cm x 6 cm was successfully patterned in the same way. Fluorescence image of the whole deposition area is shown Fig 3C as well as vertical and horizontal fluorescence intensity profiles of an enlarged section (Fig 3D).

It was found that meniscus velocities of 6.7 mm.s^-1^ for the 1cm^2^ microfluidic chamber and 13 mm.s^-1^ for the larger microfluidic chamber (inking area of 2 cm x 6 cm) were providing optimal conditions for both liquid injection and withdrawal. These meniscus velocities values were obtained for 20 μL.s^-1^ and 90 μL.s^-1^ flow rates respectively. These parameters were found to prevent meniscus breakage that may be induced due to excessive withdrawal velocities, while promoting homogeneous pattern deposition that might be hindered by evaporation effects at the three-phase contact line (also known as stick slips effects) arising at low meniscus velocities.

Finally, we observed a direct influence of the incubation time during which the flow was stopped allowing the proteins to adsorb on the stamp surface. Independently from the meniscus velocity, the duration of the inking time influences the fluorescence intensity of the printed patterns suggesting that the quantity of biomolecules deposited is higher for a longer static inking time as shown in S4 Appendix. The inking time was consequently set to 1 minute for the experiments described below.

### Multiplexed patterning

**Fig 4:**
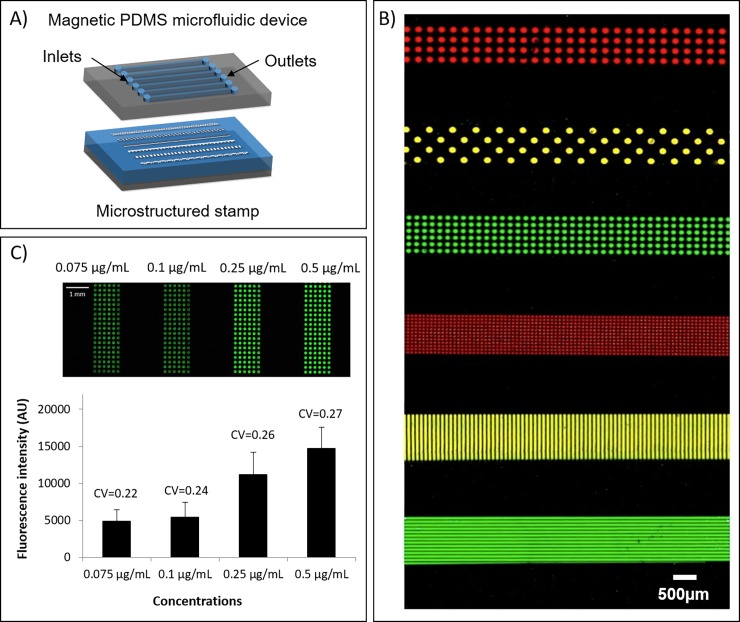
**Multiplexed protein patterning**. (A) Representative scheme of the fluidic cartridge and the structured stamp for multiplexed fluidic inking. (B) Fluorescence image of multiplexed protein deposition of Cy3-labeled streptavidin (green), Cy5-labeled streptavidin (red) and a mix of both previous solutions (yellow). Patterns dimensions were ranging from 50μm to 150μm. The image was obtained by fluorescence scanner InnoScan1100AL (PMT 532nm: 100%, PMT 635nm: 100%, Brightness: 10%, Contrast: 70%, Balance: 44%, resolution 3μm/pixel). (C) Fluorescence image after multiplexed fluidic inking and one-step printing of Cy3-labeled streptavidin with different inks of concentrations ranging from 0.075μg/ml to 0.5μg/ml.

Whereas conventional droplet-based inking technique is usually limited to the inking of a unique solution, microfluidic inking allows the parallel deposition of several solutions on the stamp followed by a one-step transfer on the substrate. We implemented a microfluidic device containing six independent channels (Fig 4A). This configuration was used as a proof of concept. It is important to notice that the number of channels can be easily increased and adapted to a larger amount of different inks. Microfluidics indeed offers the opportunity of a high level of channel integration and complexity at small length scale. The maximum number of channels is mainly limited by the size of each stamp area to be addressed and the total size of the stamp. Different solutions of fluorescently labelled streptavidin were sequentially injected in the six independent channels using the peristaltic system described above with a flow rate of 10 μL.s^-1^. Then, streptavidin patterns were simultaneously transferred on a glass slide by magnetic-assisted printing. A fluorescence image of the obtained multiplexed pattern array is shown in Fig 4B. Another substrate (Fig 4C) was successfully patterned with different concentrations of Cy3-labelled streptavidin solution (ranging from 0.075 μg.mL^-1^ to 0.5 μg.mL^-1^). As shown in Fig 4C, the mean fluorescence intensity raised by a factor 3 as the concentration was increased from 0.075 μg.ml^-1^ up to 0.5 μg.ml^-1^. Interestingly, the coefficients of variation of the measured fluorescence intensities were lower than 0.3 in the four cases with a slight increase of few percent when increasing the concentration of the inking solution. These results were not influenced either by the processed area (up to several mm^2^) or by the shapes and sizes of the patterns. The homogeneity was not affected by the proximity of the microfluidic channel walls that could eventually affect the flow velocity profile or the meniscus behavior.

### Application for cell microarray fabrication

**Fig 5:**
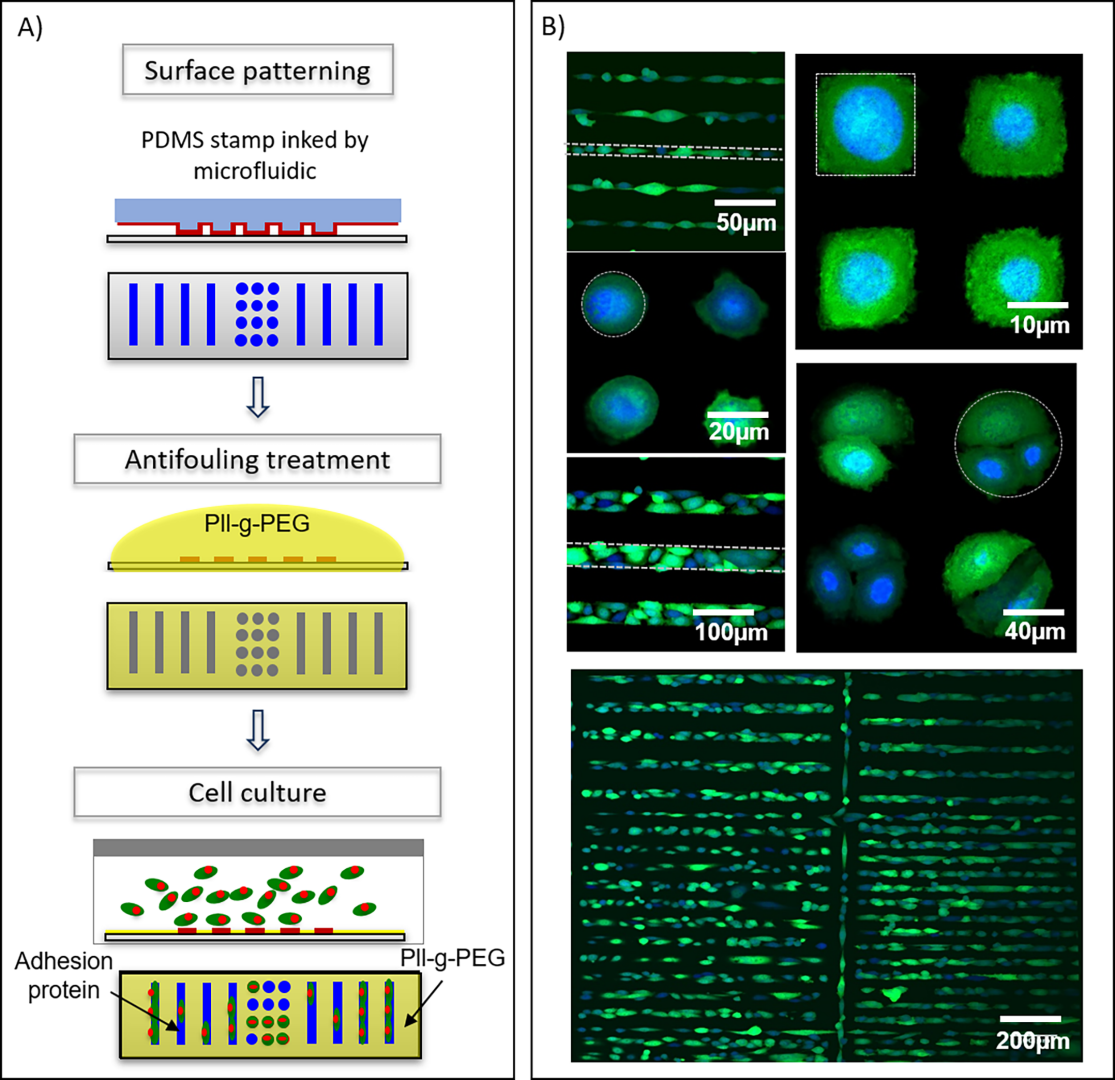
**Cell microarray fabrication**. (A) Representative scheme of cell microarray fabrication steps. (B) Fluorescence confocal images of PC3-GFP cells adhered on fibronectin patterned array. Nuclei were stained with DRAQ5™ dye (blue) and cytoplasms were expressing GFP (green). Dotted lines depict the edges of some printed features for clarity.

As a proof of concept, we evaluated the fabrication of cell microarrays. The main steps of the process are shown in Fig 5A. Briefly, a glass slide was patterned using a solution of fibronectin acting as an adhesion protein for prostate cancer cells (PC3 cell line). PLL-g-PEG was used as an anti-adhesive coating to prevent unspecific adhesion in-between the patterns. Fluorescence confocal images show PC3-GFP cells immobilized on fibronectin patterns (Fig 5B). The selectivity of the cell immobilization (calculated as the number of cells counted inside the printed patterns divided by the total number of cells counted in the probed area) was evaluated at more than 75% for each pattern shape (75% for the triangles, 85% for squares, 91% for dots and 99% for lines). Moreover, we observed that cells adapted their shape to the one of the underlying fibronectin patterns and that the number of cells grafted on a patterned feature could be controlled by adjusting the size of the pattern. Several cells were grafted on a single feature when using patterns larger than 50-μm size, whereas single cells were deterministically immobilized when using patterns of 20-μm size. Almost no cells turned out to adhere on 10-μm size patterns. These results were successfully achieved on large printed areas (> 1cm^2^) as shown in S5 Appendix.

## Conclusion

We have developed a robust approach combining magnetic forces and microfluidic devices to improve the homogeneity of the inking step of PDMS stamps used in microcontact printing. The implementation of a magnetic clamping system was particularly advantageous as it fully preserved the compatibility with most of substrate formats and dimensions. As compared to conventional inking technique, this approach significantly improved the homogeneity of biomolecule depositions on large areas up to 12 cm^2^ with a decrease of the coefficient of variation from 0.64 to < 0.3. Microfluidic inking indeed has provided an accurate and reproducible control of the critical parameters involved in the inking process, in particular the incubation time and the meniscus velocity. Beyond the reduction of sample volumes, microfluidics also offered a simple solution for multiplexed microcontact printing that can be easily combined with existing automated-commercially-available μCP systems. A first application of this homogeneous large-scale printing process for cell microarray fabrication was investigated with success. Selective cell adhesion on printed patterns was obtained over the whole surface of a glass slide, demonstrating the suitability of our procedure for high-density and large-scale cell array fabrication. This manufacturing process can thus be implemented for extensive analysis applications in biology and pharmacology such as drug screening, tissue engineering and statistically relevant fundamental studies of some cell mechanisms.

## Supporting information

S1 Appendix **Microfluidic chamber and channel designs:** (A) Design 1 for an inking area of 1cm^2^. (B) Design2 for an inking area of 12cm^2^ (whole glass slide). (C) Design 3 for multiplexed inking.
(TIF)

S2 Appendix Fabrication process of magnetic PDMS fluidic cartridge and structured stamp, and assembly. (A) Representative scheme of the two-layer fabrication process of magnetic PDMS microfluidic cartridge. (B) Representative scheme of the two-layer fabrication process of magnetic PDMS structured stamp. (C) Microfluidic device assembly with magnets.
(TIF)

S3 Appendix Comparison of protein deposition homogeneity using fluidic or conventional droplet-based inking approaches and influence of the feature position in the fluidic channel during inking. (A) Chart of fluorescence intensity values of protein depositions for both inking approaches as a function of the feature column number. (B) Fluorescence images of C3-labeled streptavidin patterned depositions obtained by μCP with fluidic or droplet-based inking approach. Images were obtained by fluorescence scanner InnoScan1100AL (PMT 532nm: 30%, Brightness: 40%, Contrast: 20%, resolution: 3μm/pixel). Scale bar: 500μm.
(TIF)

S4 Appendix **Influence of the inking time on the fluorescence intensity and homogeneity of protein depositions:** (A) Fluorescence images of Cy3-labeled streptavidin depositions for different inking times (0s, 15s, 30s and 60s) obtained with the fluorescence scanner InnoScan1100AL (PMT 532nm: 40%, Brightness: 40%, Contrast: 55%, resolution: 2μm/pixel). (B) Chart of fluorescence intensity mean values as a function of the inking time.
(TIF)

S5 Appendix Large-scale cell microarray. Fluorescence image (by fluorescence scanner InnoScan1100, excitation wavelength 532 nm) and zoomed fluorescence confocal image of PC3-GFP cells adhered on fibronectin (100 μg/ml) patterned array. Nuclei were stained with DRAQ5 dye (blue) and cytoplasms were expressing GFP (green).
(TIF)
