# Supplementary figures and images for: Dynamic inking of large-scale stamps for multiplexed microcontact printing and fabrication of cell microarrays
Source: PLoS One. 2018 Aug 23;13(8):e0202531. doi: 10.1371/journal.pone.0202531 (PMC6107178; doi:10.1371/journal.pone.0202531)

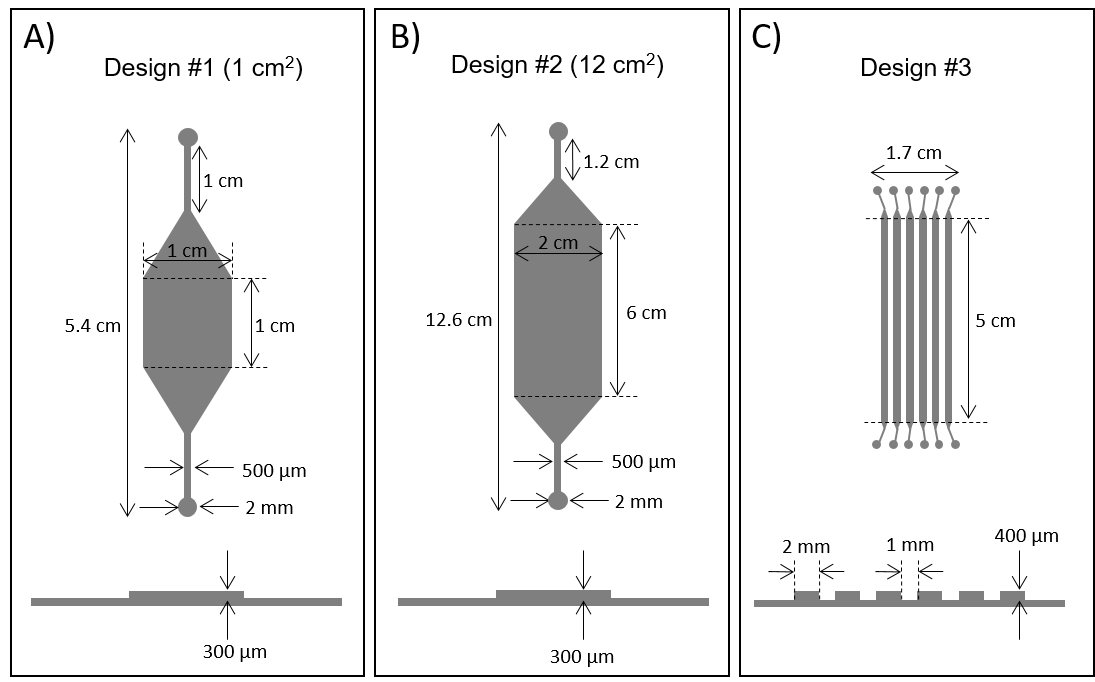

Supplement: S1 Appendix — Microfluidic chamber and channel designs: (A) Design 1 for an inking area of 1cm2. (B) Design2 for an inking area of 12cm2 (whole glass slide). (C) Design 3 for multiplexed inking. (TIF) [file pone.0202531.s001.tif]

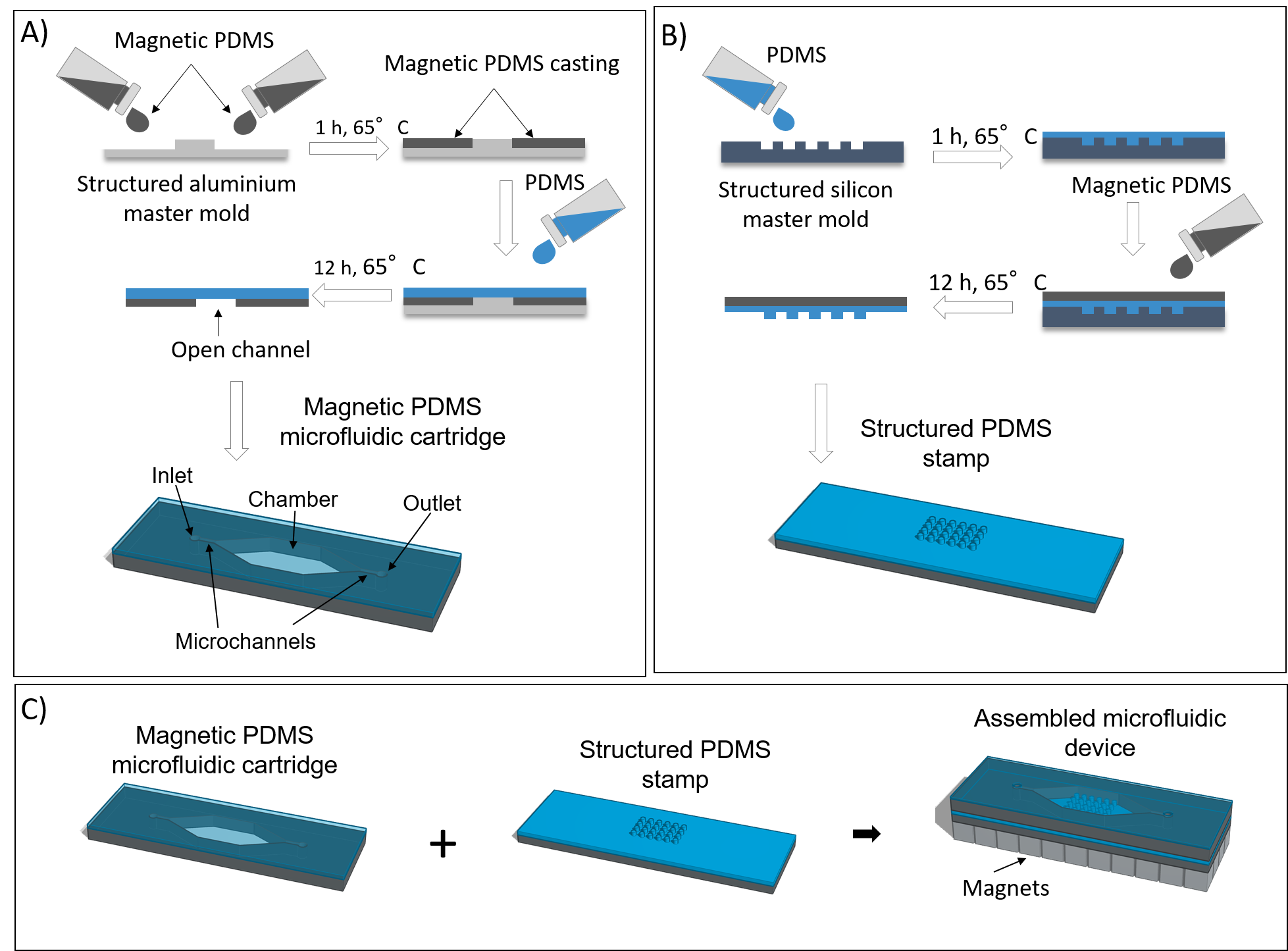

Supplement: S2 Appendix — (A) Representative scheme of the two-layer fabrication process of magnetic PDMS microfluidic cartridge. (B) Representative scheme of the two-layer fabrication process of magnetic PDMS structured stamp. (C) Microfluidic device assembly with magnets. (TIF) [file pone.0202531.s002.tif]

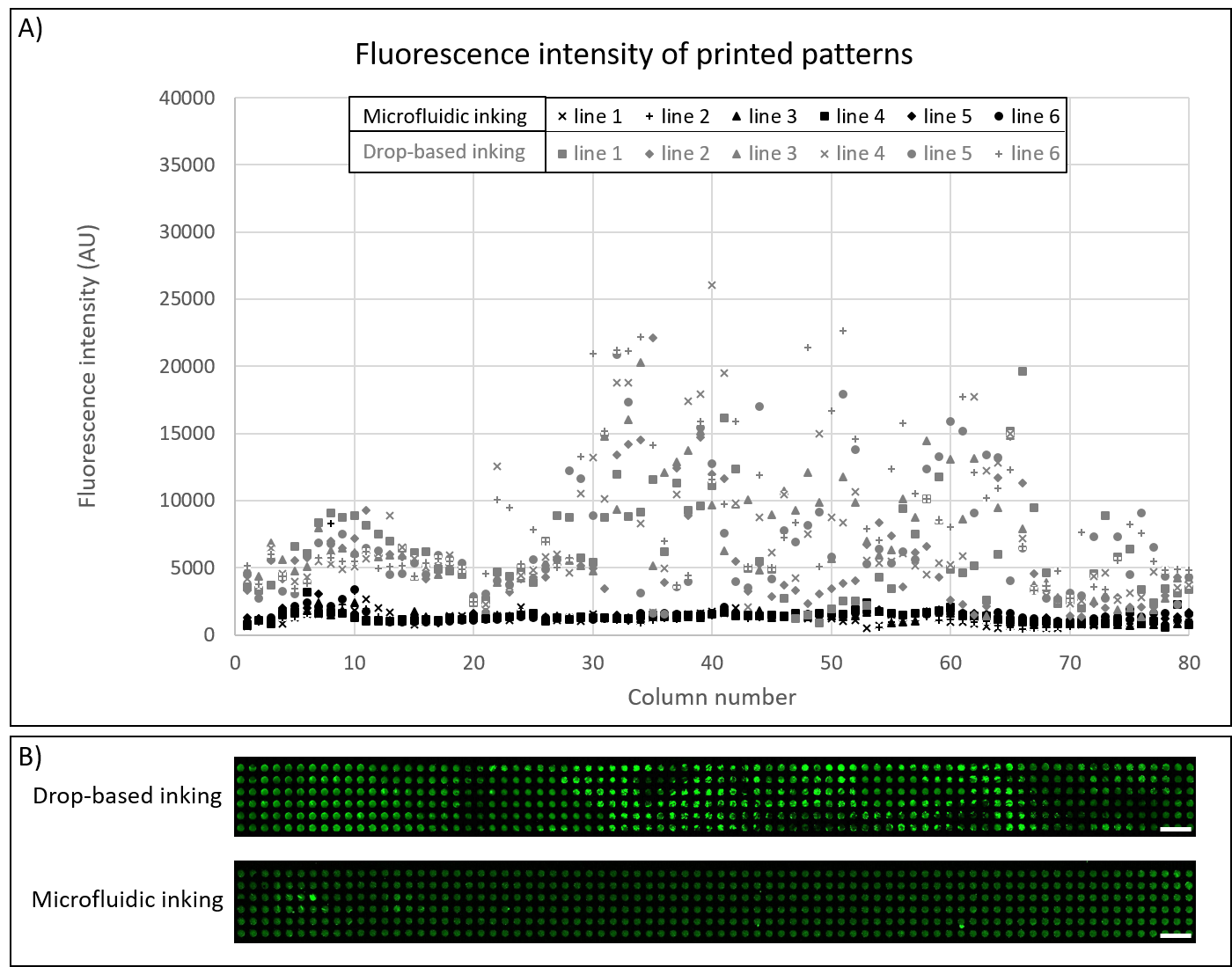

Supplement: S3 Appendix — (A) Chart of fluorescence intensity values of protein depositions for both inking approaches as a function of the feature column number. (B) Fluorescence images of C3-labeled streptavidin patterned depositions obtained by μCP with fluidic or droplet-based inking approach. Images were obtained by fluorescence scanner InnoScan1100AL (PMT 532nm: 30%, Brightness: 40%, Contrast: 20%, resolution: 3μm/pixel). Scale bar: 500μm. (TIF) [file pone.0202531.s003.tif]

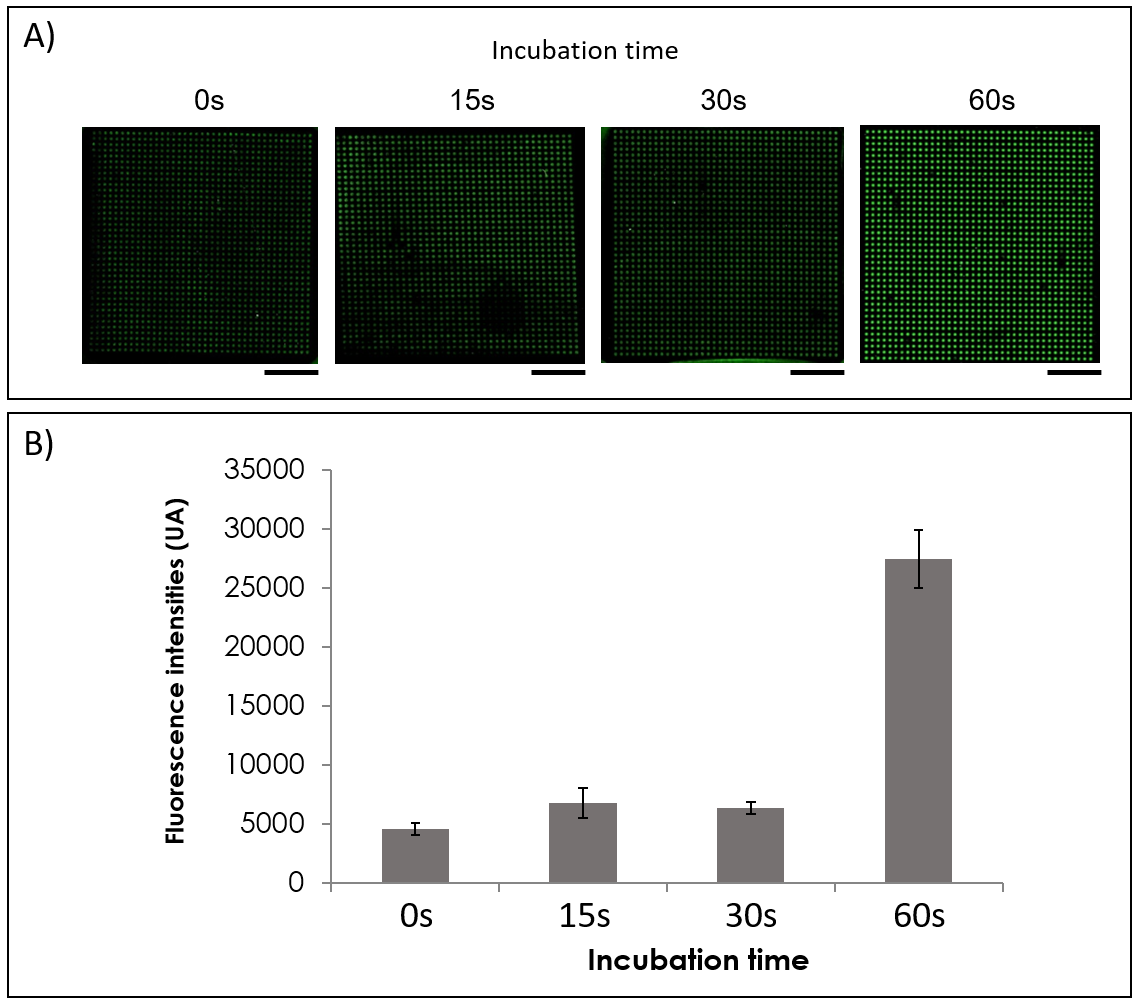

Supplement: S4 Appendix — Influence of the inking time on the fluorescence intensity and homogeneity of protein depositions: (A) Fluorescence images of Cy3-labeled streptavidin depositions for different inking times (0s, 15s, 30s and 60s) obtained with the fluorescence scanner InnoScan1100AL (PMT 532nm: 40%, Brightness: 40%, Contrast: 55%, resolution: 2μm/pixel). (B) Chart of fluorescence intensity mean values as a function of the inking time. (TIF) [file pone.0202531.s004.tif]

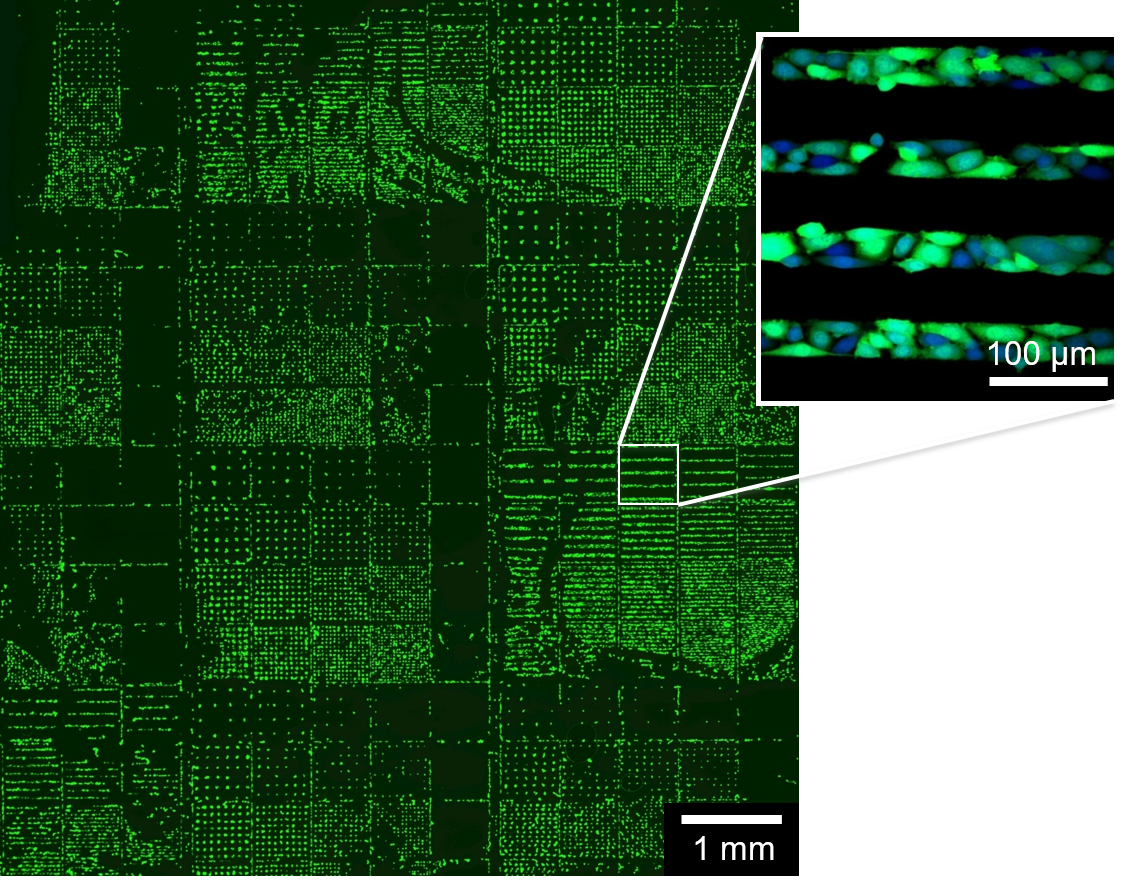

Supplement: S5 Appendix — Fluorescence image (by fluorescence scanner InnoScan1100, excitation wavelength 532 nm) and zoomed fluorescence confocal image of PC3-GFP cells adhered on fibronectin (100 μg/ml) patterned array. Nuclei were stained with DRAQ5 dye (blue) and cytoplasms were expressing GFP (green). (TIF) [file pone.0202531.s005.tif]
